# A Distinctive NAFLD Signature in Adipose Tissue from Women with Severe Obesity

**DOI:** 10.3390/ijms221910541

**Published:** 2021-09-29

**Authors:** Óscar Osorio-Conles, Arturo Vega-Beyhart, Ainitze Ibarzabal, José María Balibrea, Isabel Graupera, Jordi Rimola, Josep Vidal, Ana de Hollanda

**Affiliations:** 1 Centro de Investigación Biomédica en Red de Diabetes y Enfermedades Metabólicas Asociadas (CIBERDEM), Instituto de Salud Carlos III (ISCIII), Institut d’Investigacions Biomèdiques August Pi i Sunyer (IDIBAPS), Rosselló Street 149, 08036 Barcelona, Spain; jovidal@clinic.cat; 2 Institut d’Investigacions Biomèdiques August Pi i Sunyer (IDIBAPS), 08036 Barcelona, Spain; BEYHART@clinic.cat (A.V.-B.); jrimola@clinic.cat (J.R.); amdehol@clinic.cat (A.d.H.); 3 Gastrointestinal Surgery Department, Hospital Clínic de Barcelona, 08036 Barcelona, Spain; aibarza@clinic.cat (A.I.); BALIBREA@clinic.cat (J.M.B.); 4 Centro de Investigación Biomédica en Red de Enfermedades Hepáticas y Digestivas (CIBERehd), Liver Unit, Hospital Clínic de Barcelona, 08036 Barcelona, Spain; igraupe@clinic.cat; 5 Obesity Unit, Endocrinology and Nutrition Department, Hospital Clínic de Barcelona, 08036 Barcelona, Spain; 6 Centro de Investigación Biomédica en Red Fisiopatologia de la Obesidad y Nutrición (CIBEROBN), Instituto de Salud Carlos III (ISCIII), 28029 Madrid, Spain

**Keywords:** NAFLD, obesity, adipose tissue

## Abstract

Development and severity of nonalcoholic fatty liver disease (NAFLD) have been linked to obesity and white adipose tissue (WAT) dysfunction plays a key role in this relation. We compared the main features of subcutaneous (SAT) and visceral WAT (VAT) tissue dysfunction in 48 obese women without (Ob) and with NAFLD (Ob-NAFLD) undergoing bariatric surgery and matched for age, BMI and T2D status. Fat cell area, adipocyte size distribution, the degree of histological fibrosis and the mRNA expression of adipokines and genes implicated in inflammation, adipogenesis, angiogenesis, metabolism and extracellular matrix remodeling were measured by RT-qPCR in both fat depots. Ob-NAFLD group showed higher TG and lower HDL circulating levels, increased VAT fat cell area and similar WAT fibrosis in comparison with Ob group. A sPLS-DA was performed in order to identify the set of genes that better characterize the presence of NAFLD. Finally, we build a multinomial logistic model including seven genes that explained 100% of the variance in NAFLD and correctly predicted 100% of cases. Our data support the existence of distinctive NAFLD signatures in WAT from women with severe obesity. A better understanding of these pathways may help in future strategies for the prevention and treatment of NAFLD.

## 1. Introduction

NAFLD is defined as the presence of >5% of hepatic steatosis in the absence of other competing liver disease etiologies, concomitant use of alcohol or therapies that may induce liver steatosis [1]. NAFLD is the most prevalent chronic liver disease worldwide, affecting between 25–30% of the general population [2]. A “multiple hit” hypothesis has been proposed at the origin of its pathophysiology. Accordingly, multiple insults act together on genetically predisposed subjects to induce NAFLD [3]. These multiple parallel “hits” induce chronic systemic inflammatory processes altering key metabolic tissues, although complete portray of the factors that contribute to disease progression remain partially understood [4]. NAFLD patients tend to be obese and have other metabolic syndrome comorbidities. Thus, obesity is established as a contributor to the initial process leading to simple steatosis [5], indeed, more than 90% of severely obese patients that undergo bariatric surgery present with NAFLD [1]. However, NAFLD can also develop in subjects with normal body mass index (BMI) suggesting that metabolic alterations observed in obese patients that are found in other conditions might be drivers of NAFLD [6]. In fact, multiple studies have found that adipose tissue dysfunctions such as increased flux of free fatty acids (FFAs) to the liver, de novo hepatic lipogenesis and the release of pro-inflammatory cytokines by white adipose tissue (WAT) macrophages play principal roles in NAFLD development [7,8,9].

WAT expansion associated with body fat accrual leading to obesity, is accompanied by pro-inflammatory macrophage recruitment [10,11] which shifts cytokines production towards a more steatogenic, and fibrogenic profile. Indeed, it has been shown that the obesity-associated dysfunction in both subcutaneous (SAT) and visceral WAT (VAT) are responsible of up to 50% of circulating IL-6 secretion, contributing to systemic chronic inflammation [5]. Data on the importance of VAT in the chronic inflammation that drives NAFLD demonstrates that the lack of adipocyte AMP-activated protein kinase worsens liver disease by affecting brown and beige adipose tissue function [12].

WAT dysfunction is present in almost all patients with severe obesity and highly associated with NAFLD prevalence [13]. Nevertheless, few studies have systematically compared obesity-associated WAT dysfunction in relation to NAFLD condition. While early studies pointed to VAT depot as a main independent predictor of hepatic steatosis [14], liver inflammation and fibrosis [15], later investigations emphasized the association between NAFLD and SAT transcriptome [16,17,18] or macrophage phenotype [18]. Thus, du Plessis et al. built a predictive model to determine patients’ liver histology based on SAT and VAT gene expression and found a model of 5 SAT genes as the most predictive in accurately assigning patients to histological groups. Results from this study underlined the importance of the SAT gene set at both early stages of NAFLD and in its progression to steatohepatitis [16]. Nevertheless, study groups were not matched for sex or central obesity. Recently, Song et al. constructed a transcriptomic signature in WAT which showed good performance in diagnosing NAFLD [19]. However, this model was based only on immune-related genes and the depot origin of the WAT samples was not ascertained. Furthermore, none of these studies evaluated the degree of WAT fibrosis or fat cell size distribution. Therefore, we aimed to assess VAT and SAT histologic and transcriptomic differences of female patients with severe obesity and NAFLD compared to matched females for age, BMI and T2D status.

## 2. Results

The clinical characteristics of the 48 female participants are shown in Table 1. One-to-one propensity score matching (PSM) methodology was used to obtain 24 obese women without (Ob) and 24 with NAFLD (Ob-NAFLD) pair-matched for age, BMI and T2D status, amongst subjects undergoing bariatric surgery between 2019 and 2021 at our Institution. After matching, serum triglycerides (TG) were higher and high-density lipoprotein (HDL) cholesterol levels were lower in the Ob-NAFLD group. In addition, two biochemical indices of liver fibrosis were calculated. Both FIB-4 and APRI (AST to platelet ratio index) scores were similar between groups and had low values, indicating great negative predictive value for advanced fibrosis.

### 2.1. Adipose Tissue Morphology

**Figure 1:**
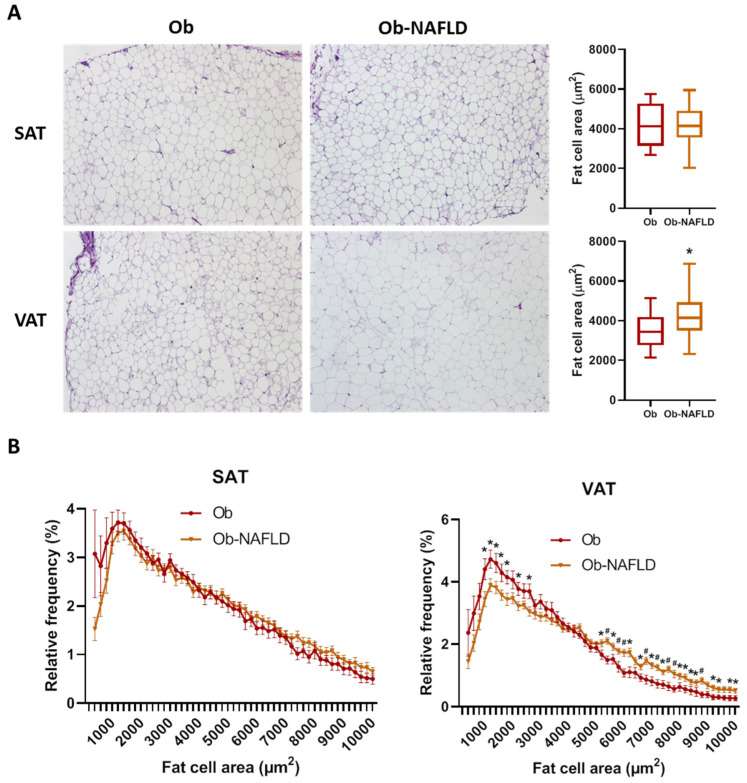
Fat cell size distribution. (**A**) Comparison of adipocyte mean cell surface area and representative images of SAT and VAT samples from Ob and Ob-NAFLD individuals. (**B**) Frequency distribution analysis of fat cell areas divided by size into bin intervals of 200 µm2. Data are presented as mean ± SD frequencies of cells within each bin and compared by Mann-Whitney U test. SAT, subcutaneous adipose tissue; VAT, visceral adipose tissue. * = *p* < 0.05, ^#^ = *p* < 0.01.

Histological analysis of adipocyte area and tissue fibrosis was performed on formalin-fixed sections from both fat depots. Mean SAT-adipocyte area did not significantly differ among groups (Table 2, Figure 1A). On the contrary, mean VAT-adipocyte area was increased by 20% in the Ob-NAFLD group (Table 2, Figure 1A). Analysis of the adipocyte size distribution revealed that NAFLD condition is associated with a lower abundance (16% less) of smaller (<3000 µm^2^) and higher abundance (55% more) of larger adipocytes (> 6000 µm^2^) in VAT relative to Ob (Figure 1B), while no significant differences were found in SAT. Furthermore, the mean ratio between individual SAT and VAT fat cell area was significantly lower in the Ob-NAFLD group (Table 2). Sirius red staining showed that fibrosis around adipocytes (i.e., pericellular fibrosis) were similar in both depots irrespective of NAFLD condition (Table 2, Supplementary Figure S1).

### 2.2. Differential Gene Expression Analysis

Descriptive statistics of the expression analysis of 75 genes implicated in WAT dysfunction evaluated both in SAT and VAT, grouped by NAFLD status are shown in Supplementary Table S1. The expression of 15 genes in SAT and eight genes in VAT was significantly different in Ob-NAFLD versus Ob subjects. Relative expression levels and fold changes between groups are reported in Table 3.

In SAT, the pan-macrophage marker CD68 and the M2-type macrophage markers MSR1/CD204 and MRC1/CD206 were found upregulated in patients with NAFLD (Table 3, Supplementary Figure S2). The expression of vascular endothelial growth factor B (VEGFB) and angiopoietin 1 (ANGPT1), two pro-angiogenic factors was also increased in the Ob-NAFLD group by 38% and 41%, respectively. In addition, leptin (LEP) gene expression was up- while its receptor (LEPR) was downregulated. Several genes implicated in ECM composition and remodeling were found altered in the NAFLD group. Thus, despite similar levels of histological fibrosis, TGFB1 gene expression was upregulated by 70%. Similarly, the expression of the α-1 chains of collagens I, IV and VI were up-modulated, especially COL6A1 (by 940%). On the contrary, the tissue inhibitor of metalloproteinases-3 (TIMP3) was down-regulated by 48%.

In VAT, the expression of hypoxia-inducible factor 1-alpha (HIF1A), ANGPT2 and the thermoregulatory gene cell death-inducing DFFA-like effector A (CIDEA) was found slightly decreased in NAFLD obese females (Table 3, Supplementary Figure S3). The expression of senescence genes TP53/P53 and CDKN2A/P16 was decreased (by 49 and 35%, respectively) while LEP and monoglyceride lipase (MGLL) were significantly upregulated (by 87 and 67%, respectively).

Of note, both fat depots showed comparable fold changes of the senescence marker P16 (0.3-fold) and LEP (1.8-fold) in NAFLD patients. Finally, the ratio between LEP and ADIPOQ expression was increased in VAT of Ob-NAFLD patients (0.20 (0.10–0.41)) with respect to OB (0.10 (0.06–0.17), *p* = 0.046) while comparable in SAT from both groups (0.25 (0.14–0.33) vs. 0.27 (0.12–0.35), *p* = 0.696).

### 2.3. Principal Component Analysis (PCA) to Identify NAFLD Patients

**Figure 2:**
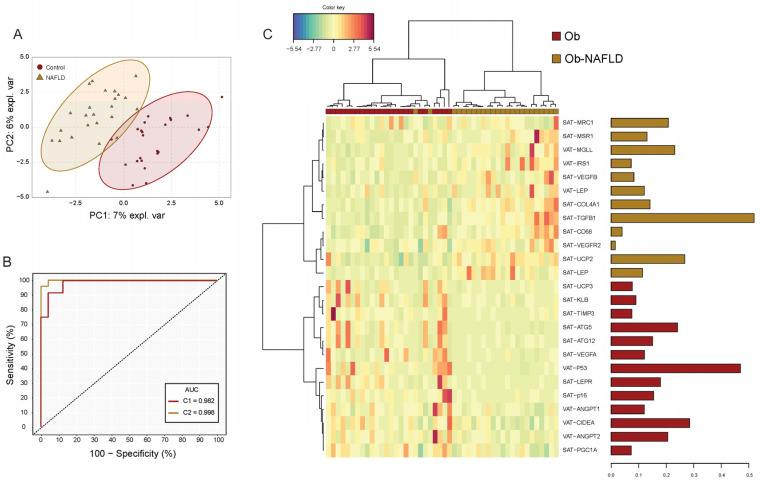
Subcutaneous and visceral fat gene expression signature model for NAFLD. (**A**) Sparse partial least square discriminant analysis (sPLSDA) individual scatter score plot (X-axis: component 1 = 25 genes; Y-axis: component 2 = 25 genes). Ellipses shades indicate 95% CI. (**B**) Receiver operating characteristic (ROC) analysis of the gene expression signature model. Red line corresponds to the accuracy classification performance of component 1-genes whereas yellow line to the model composed by genes (*n* = 50) of both components. (**C**) Hierarchical dendrogram-heatmap of the genes conforming sPLSDA Component 1. Log2 transformation is performed on the mRNA raw values of each gene. Complete linkage clustering method with Euclidean distance are used. The contribution of each gene for the model is represented in the barplot where each bar length corresponds to the loading weight (importance) of each variable. Bar color indicates the group in which the mean gene expression is greater.

The PCA including expression data of all 150 genes did not spot defined cluster differences between patients with and without NAFLD (Q2X = 21.9%5, R2X = 45.3%) (Supplementary Figure S4). In order to find an adipose tissue-associated genomic signature capable to differentiate patients with NAFLD, a sPLS-DA model cross-validated via LOO (R2Y = 0.94; Q2 = 0.43, *p* = < 0.001) (Figure 2A) was tuned. The sPLS-DA signature composed by 25 genes in C1 was the most accurate model capable of classifying patients with NAFLD with an area under the ROC curve of 0.982 (BER = 0.118, *p* < 0.001) (Figure 2B). The signature heatmap showed a clear hierarchical division between genes associated with the presence of NAFLD (Figure 2C). Among the signature-genes highly expressed in patients with NAFLD, SAT−TGFB1, SAT−UCP2, VAT−MGLL, SAT−MRC1 and SAT−COL4A1 were the top 5 genes which contributed the most to the model. On the other hand, VAT−P53, VAT−CIDEA, SAT−ATG5, VAT−ANGPT2 and SAT−LEPR were those genes down regulated with larger contribution (Supplementary Table S1). All genes from the signature model had mean expressions significantly different in patients with NAFLD.

Multinomial logistic regressions were performed to assess the effects of SAT and VAT gene expression on the likelihood that patients have NAFLD (Table 4). SAT-TGFB1 and VAT-P53 resulted the only genes independently associated with the presence of NAFLD. Patients with higher expressions of SAT-TGFB1 were 4.04 (1.15–14–23) times more likely to present NAFLD whereas increasing VAT-P53 expression was associated with a 0.25 likelihood reduction (0.07–0.85) of having the disease. A model including these genes explained 45.2% of the variance in NAFLD and correctly classified 80.0% of cases (*p* = 0.000). Moreover, we found a second model including 7 genes (Table 4) that explained 100% of the NAFLD variance (*p* = 0.000) though no gene reached independent significance explained because of gene intercorrelation (Supplementary Table S2)

## 3. Discussion

In the present study, we characterized the main histological and gene expression differences in SAT and VAT between females with severe obesity without and with NAFLD as assessed from hepatic ultrasound. Through matching patients by age, BMI and T2D, WAT alterations induced by NAFLD per se could be evaluated avoiding potential confounders. Overall, anatomical changes were mainly found in VAT adipocyte area of NAFLD patients with a shift in the proportion of adipocytes to a larger fat cell area range. However, in transcriptomic analysis, both fat compartments had differentially expressed genes associated with NAFLD, which constituted a highly discriminant WAT-signature of 25 genes. Finally, SAT-TGFB1 and VAT-P53 expression were genes with high likelihood estimates independently associated with NAFLD.

Clinically, obesity associates increased morbidity and mortality when combined with NAFLD. Pathogenetically, obesity and the ensuing insulin resistance contribute to the initial fat accumulation in the hepatocyte and to its progression into nonalcoholic steatohepatitis (NASH), NASH-related cirrhosis and hepatocellular carcinoma [20]. Nevertheless, obesity does not always concur with NAFLD, as not all NAFLD patients are obese and not all subjects living with obesity present NAFLD [21,22]. Different causal links have been described to explain the association between obesity and NAFLD [23,24,25], and altered adipose tissue biology has been recognized as a key early event in its initiation. However, to our knowledge, the number of studies systematically comparing the biology of WAT in relation to the presence of NAFLD is very limited to date [16,26,27], comparisons are made with respect to healthy lean controls [28] or are focused on specific WAT-derived circulating factors [29,30].

Obesity-induced changes in adipokine secretion can modify the adipose tissue-liver crosstalk [31,32] and the ratio between leptin and adiponectin has been proposed as a superior biomarker of NAFLD than adiponectin or leptin measurements alone [33,34]. Here we report higher SAT and VAT leptin expression in NAFLD group while comparable levels of adiponectin expression, what translates into increased visceral leptin-to-adiponectin ratio in this group. Greater leptin expression in both fat depots contributed to classify patients with NAFLD in the sPLS-DA, although it was not included in the multinomial logistic regression model.

The ‘portal theory’ suggested that, in obese patients, venous drainage of increasing amounts of pro-inflammatory factors and free fatty acids from VAT to the liver via the portal system favors the development of hepatic insulin resistance and liver steatosis [35,36,37]. However, some authors presented evidence challenging this view and questioned its relative contribution with respect to SAT [38]. Despite VAT dysfunction is generally considered as the main contributor to NAFLD [14,15], SAT expression of genes was overrepresented in all group comparisons in our study, being 15 vs. 8 the number of genes in SAT vs. VAT, respectively, whose expression was significantly different in the two sample comparisons; 18 vs. 7 were included in the sPLS-DA model and 4 vs. 3 were included in the multinomial logistic regression model. However, this does not imply a direct causal relationship of SAT dysfunction in the development of NAFLD and the design of this study prevents drawing mechanistic conclusions. One possibility is that greater SAT dysfunction leads to greater VAT dysfunction, and this in turn is associated with the development of the NAFLD.

In a multicenter study, du Plessis et al. built a predictive model of NAFLD based on SAT and VAT gene expression levels from severely obese patients [16]. Interestingly, their results emphasize the significance of SAT inflammation and the highly predictive nature of the SAT gene set in classifying liver histology over VAT. In this sense, previous studies described that SAT macrophages resembled the pro-inflammatory phenotype of VAT macrophages and were significantly increased in patients with NASH and fibrosis [18]. Similarly, the transcription of pro-inflammatory genes and macrophage numbers in SAT correlated with hepatic fat content in other study [17].

Our model including SAT expression of TGFB1 and VAT expression of P53, two tumor suppressor genes, correctly classified 80.0% of NAFLD cases. TGFB1, which was upregulated in Ob-NAFLD group, promotes the release of inflammation mediators, remodeling and collagen deposition in WAT [39,40,41]. TGFB1 release from SAT is increased in obesity [42] while systemic blockade of its signaling protects mice from obesity, diabetes and hepatic steatosis [43].

Accumulating evidence suggests that the role of P53 in the progression of NAFLD is complex and context-dependent [44]. P53 plays multiple functions in cell cycle, cellular senescence, autophagy, apoptosis, and metabolism [45]. While hepatic p53 expression is elevated in patients with NASH [46,47] and positively correlates with the degree of steatosis [47], WAT p53 seems to play a specific role as a negative regulator of adipogenesis [45] and reduces lipid droplet accumulation [48]. In our study, visceral expression of P53 was reduced in NAFLD patients but, strikingly, this was accompanied by a higher adipocyte hypertrophy.

Interestingly, the expression of CIDEA, which was found downregulated in VAT and main predictor of NAFLD, has been positively associated with human obesity but inversely related to NAFLD severity [49]. CIDEA is part of the cell death-inducing like effector (CIDE) family that regulates hepatic lipid homeostasis controlling lipid droplet growth. The downregulation of VAT-CIDEA found in our NAFLD population points out one of the genes that might be independently associated to liver injury irrespective from obesity.

SAT-UCP2 which was highly increased in patients with NAFLD and was the third gene in the signature contributors, is a mitochondrial anion carrier which expression has been associated to cells that are exposed to obesity-associated oxidative stress as a defensive mechanism [50]. UCP2 overexpression has been found to induce acute liver injury and HFD-induced liver damage while its downregulation its associated with NAFLD amelioration in animal models [51].

MGLL, whose visceral expression was increased in patients with NAFLD, its part of the monoglyceride lipases family that hydrolyzes triglycerides to fatty acids and glycerol in VAT [52] and may indicate an increased lipolysis in the NAFLD group, leading to excessive circulating FFA levels and contributing to the development of NAFLD.

We report a decreased expression of the autophagy related gene 5 (ATG5) in SAT from NAFLD patients. While pharmacological inhibition, silencing or knockdown of ATG5 in hepatocytes results in increased triglyceride levels and lipid droplet accumulation [53], WAT expression of ATG5 is increased in obesity, especially in the VAT depot [54], and is a key regulator of adipogenesis. In mature adipocytes, however, is still not clear the role of autophagy and if it is beneficial or detrimental [55].

In contrast with our results of VAT-ANGPT2 being downregulated in patients with NAFLD, a study of 93 severely obese subjects found that ANGPT2 expression in VAT was associated with different NAFLD features, including steatosis, ballooning, portal and lobular inflammation [56]. However, this correlation was lost after correction for T2D and insulin resistance and plasma ANGPT2 levels were not related to NAFLD features. Moreover, the study did not include a control group without NAFLD.

Finally, we found an upregulation of mannose receptor C-type 1 (MRC1) in SAT from the NAFLD group. MRC1, a surface marker of M2 or anti-inflammatory macrophages, was previously found increased in SAT from obese subjects with NAFLD versus obese with normal intrahepatic triglyceride content and lean control patients [57].

We acknowledge our study is not without limitations. First, diagnosis of NAFLD was based only on ultrasound assessment, and not in the gold standard liver biopsy. Thus, presence of mild liver steatosis could not be ruled out in our study subjects classified in the non-NAFLD group. Although liver biopsies were not performed, data from validated scores would suggest our study patients were at early stages of NAFLD. Second, even though the groups were paired for BMI and T2D, body composition and fat distribution of the participants were not evaluated. Finally, the approach presented here, despite highly discriminant to diagnose NAFLD, would not be a more feasible alternative than the gold standard liver biopsy nor than non-invasive tests such as biomarkers or ultrasound, since a VAT sample would be still needed.

Overall, within its limitations, our data support the existence of a distinctive NAFLD-associated histological and transcriptomic signature in WAT from females with severe obesity even when compared to patients with similar clinical parameters. Further studies are needed to confirm our data, as well as functional studies are warranted to establish causal relationships between these genes and the development of NAFLD. A better understanding of this pathways may help in future strategies for the prevention and treatment of NAFLD.

## 4. Materials and Methods

### 4.1. Study Population

Forty-eight severely obese women were selected among candidates undergoing bariatric surgery at the Obesity Unit of the Hospital Clínic of Barcelona. The study’s exclusion criteria were a history of malignancy, chronic inflammatory diseases, active infectious diseases, drug abuse or daily alcohol consumption >20 g. Twelve patients underwent a laparoscopic sleeve gastrectomy and thirty-six underwent a gastric bypass. Two matched female cohorts, matching NAFLD 1:1 to non NALFD subjects (24 vs. 24), were obtained using “nearest neighbor” matching for age, BMI and T2D and the maximum allowed distance was a Δ of 0.001 (SPSS, version 25.0, Chicago, IL, USA). The matching significantly reduced differences in these variables. Ethics committee approval conforming to the Declaration of Helsinki was obtained from the Clinical Research Ethics Committee (CEIC) of Hospital Clinic de Barcelona. All participants provided written informed consent.

### 4.2. Clinical and Anthropometric Data

The patients’ anthropometric measurements were collected in the same consultations following standardized procedures and hematological and biochemical parameters were determined at the Core Laboratory of the Biomedical Diagnostic Center using an Advia 2400 analyzer (Siemens Healthcare S.L.U., Getafe, Spain). Anthropometric and clinical data are summarized in Table 1.

### 4.3. Determination of NAFLD

Presence of NAFLD was ascertained as part of the pre-bariatric surgery evaluation and following exclusion of secondary causes of liver steatosis and excessive alcohol intake (defined as ≥20 g/d) [58]. NAFLD was defined as the presence of hepatic steatosis on ultrasonography (US). Hepatic US was performed in all patients after 6 h fasting, by a single experienced using a clinical US scanner (Aplio i-800, Canon Medical Systems S.A., Madrid, Spain) equipped with a i8CX1 1–8-MHz curved US transducer used for conventional B-mode examination. Each subject was examined in the supine and left lateral positions during quiet inspiration. Hepatic steatosis was diagnosed on the basis of characteristic US features: evidence of diffuse hyperechogenicity of the liver relative to the kidneys, ultrasound beam attenuation and poor visualization of intra-hepatic vessel borders and diaphragm. Semiquantitative US scoring for the degree of hepatic steatosis was not available in this study. Absence of advanced liver fibrosis was evaluated using the fibrosis-4 (FIB-4) and the AST to platelet ratio index (APRI) according to the proposed cut-offs [58].

### 4.4. White Adipose Tissue Biopsies

Paired SAT and VAT samples were obtained at the time of surgery. Samples were collected in DMEM and rinsed in PBS. A portion was immediately frozen before RNA analysis. Other portion was fixed overnight at 4 °C in 4% paraformaldehyde and processed for standard paraffin embedding. Starting at the tissue apex 3 × 3 μm sections were made at a minimum of 100 μm intervals across the sample tissue. Serial sections were matched for additional independent analyses.

### 4.5. Morphometry and Histopathology

Hematoxylin and eosin (H&E) staining was used to assess adipocyte morphology. Digital images were captured under an X600 microscope (Olympus, Tokyo, Japan) at 4× magnification. Adipocyte size of at least 3000 cells per sample was measured within a minimum of 5 micrographs from randomly selected fields using Adipocytes Tools, an ImageJ macro-based algorithm for ImageJ software (National Institutes of Health, Bethesda, MD, USA; http://imagej.nih.gov/ij/, last accessed 20 August 2021). Adipocyte average area was calculated and frequency distribution analysis into bin intervals of 200 µm^2^ was performed.

Sirius red staining was used for quantification of pericellular fibrosis (i.e., extracellular matrix accumulation around the cells). Automated analysis of at least 10 images per sample at 10× magnification has been carried out using MRI Fibrosis Tool, an ImageJ macro-based algorithm, and expressed as a percentage of red staining (fibrosis)/tissue surface ratio. SAT:VAT fibrosis ratio has been calculated as the ratio between the percentage of fibrotic area in SAT and VAT from each patient.

### 4.6. RNA Extraction and Real Time PCR

Total RNA was isolated using RNeasy Lipid Tissue Mini Kit (Qiagen, Hilden, Germany). Concentration and purity were measured using a NanoDrop 1000 spectrophotometer (Thermo Scientific, Waltham, MA, USA). Equal amounts of RNA from SAT and VAT (2 μg) were reverse-transcribed using the Superscript III RT kit and random hexamer primers (Invitrogen, Carlsbad, CA, USA). Reverse transcription reaction was carried out for 90 min at 50 °C and an additional 10 min at 55 °C. An expression analysis of 75 genes involved in WAT dysfunction and related to inflammation, adipogenesis, autophagy, fatty acid metabolism and oxidation, adipocyte brightening, glucose metabolism and adipokines was performed in both fat depots. Real time quantitative PCR (qPCR) was performed with a 7900HT Fast Real-Time PCR System (Applied Biosystems, Foster City, CA, USA) using GoTaq^®^ qPCR Master Mix (Promega Biotech Ibérica, Madrid, Spain). Expression relative to the housekeeping gene RPL6 was calculated using the delta Ct (DCt) method. Gene expression is presented as the 2^(-DCt) values. Fold changes were calculated as the ratio between the mean expression levels in Ob-NAFLD subjects and Ob subjects. The list of primers used in this study is provided in Supplementary Table S4.

### 4.7. Statistics

Continuous data with normal and non-normal distribution is expressed with arithmetic means and standard deviations (SD), or medians and interquartile ranges (IQR), respectively. Categorical variables are expressed with frequencies and proportions. No data transformation was performed on gene expression when performing univariate analysis and normality assumption was tested with Shapiro–Wilk test. Linearity, and absence of multicollinearity were also checked. For each gene, the magnitude of the expression-difference between groups was calculated with mean fold changes (FC) and tested with Mann–Whitney U test, Welch’s *t*-test or Student’s *t*-test when adequate.

Multivariate dimensionality reduction methods were conducted to build a predictive classification model for NAFLD. First, missing gene expression values (*n* = 886, 12.02%) were imputed with the k-nearest neighbor algorithm and standardized to zero means and unit variances giving similar importance to all genes independently to its intrinsic tissue expression. Principal component analysis (PCA) using singular value decomposition was implemented to examine the intrinsic dimension and visualize the general structure of the transcriptome across groups. To find the most suitable signature, leave-one-out cross-validation of a sparse partial least square discriminant analysis (PLS-DA) was used to determine the optimal number of components and genes to be included in the model. The number of components was chosen based on the estimation of the lowest balanced error rate (BER) and the genes based on the lowest prediction error for each subset of genes on each component. R2Y (the sum of squares) and Q2Y (the predictive performance) values were assessed to ensure the absence of overfitting of the final model. Area under the curve (AUC) values were calculated from the predicted scores in the LOO cross-validation process minimizing the risk of overfitting. A clustered image map was created employing multivariate Euclidean distance metric with complete linkage method and presented with associated dendrograms. Finally, Spearman correlations followed by polynomial regressions with enter method were then performed to identify genes independently associated with the presence of NAFLD among those included in the model. Correction by age adjustment was used in all multivariable models.

All the comparisons stated as different in the present manuscript have statistical significance with a two-tailed *p*-value < 0.05. GraphPad PRISM (GraphPad Software, version 6.0, San Diego, CA, USA), Statistical Package for Social Sciences software (SPSS, version 25.0, Chicago, IL, USA) and R (The R Project for Statistical Computing, version 4.1, Vienna, Austria) software environment [59] were used to perform the analyses.

## Figures and Tables

**Table 1:** Anthropometric and clinical characteristics of the study patients.

	Ob (*n* = 24)	Ob-NAFLD (*n* = 24)	*p*-Value
Age (years) ^a^	44.88 ± 10.87	45.75 ± 8.94	0.762
BMI (kg/m2) ^a^	44.00 ± 5.32	44.41 ± 4.75	0.778
Waist (cm)	118.5 (111.3–129.3)	119.5 (116.3–125.8)	0.900
Hip (cm)	137.7 ± 9.49	133.2 ± 9.145	0.222
Waist-to-hip ratio	0.88 ± 0.08	0.91 ± 0.05	0.246
CUN-BAE Index	54.2 (52.3–55.6)	54.5 (52.7–56.5)	0.660
HTN	5 (20.83%)	9 (37.50%)	0.341 ^b^
TG (mg/dL)	189.0 ± 33.02	200.0 ± 37.96	0.027
Total cholesterol (mg/dL)	193.82 ± 24.44	202.55 ± 36.2	0.289
HDL (mg/dL)	53.75 ± 8.65	45.58 ± 7.88	0.001
LDL (mg/dL)	116.3 ± 25.08	125.2 ± 29.96	0.277
FPG (mg/dL)	90.5 (88.5–101.0)	99.0 (90.5–105.8)	0.264
T2D ^a^	5 (20.83%)	5 (20.83%)	>0.999 ^b^
HbA1c (%)	5.5 (5.3–5.7)	5.6 (5.3–6.1)	0.493
AST (IU/L)	20.5 (16.7–23.0)	21.0 (17.0–27.7)	0.454
ALT (IU/L)	21.0 (15.0–25.0)	22.0 (17.2–40.5)	0.159
GGT (IU/L)	19.0 (14.0–31.0)	24.0 (18.2–38.7)	0.349
AST:ALT ratio	1.0 (0.8–1.1)	0.8 (0.6–1.0)	0.158
Platelets (×10^9^/L)	268 (241–306)	294 (248–382)	0.125
CRP (mg/dL)	0.48 (0.34–1.21)	0.95 (0.38–1.81)	0.230
FIB-4 Score	0.71 (0.51–0.86)	0.69 (0.44–0.91)	0.778
APRI Score	0.18 (0.14–0.22)	0.19 (0.14–0.21)	0.813

Data are presented mean ± SD, median (IQR) or *n* (%). NAFLD, nonalcoholic fatty liver disease; BMI, body mass index; CUN-BAE Index, body adiposity estimator; HTN, hypertension; TG, serum triglyceride level; HDL, serum high-density lipoprotein cholesterol level; LDL, serum low density lipoprotein cholesterol level; FPG, fasting plasma glucose; T2D, type 2 diabetes; HbA1c, glycosylated haemoglobin; AST, serum aspartate aminotransferase level; ALT, serum alanine aminotransferase level; GGT, gamma-glutamyl transferase; CRP, C-reactive protein; FIB-4, index for liver fibrosis; APRI, AST to platelet ratio index. Welch’s *t*-test or Mann-Whitney test were applied except otherwise stated. ^a^ Matching variable. ^b^ Fisher’s exact test.

**Table 2:** Histological characteristics of WAT from Ob and Ob-NAFLD groups.

	Ob (*n* = 24)	Ob-NAFLD (*n* = 24)	*p*-Value
SAT fat cell area (µm^2^)	4132 ± 1124	4260 ± 985	0.728
VAT fat cell area (µm^2^)	3475 ± 851	4174 ± 992	0.038
SAT:VAT fat cell area ratio	1.25 ± 0.33	1.03 ± 0.18	0.017
SAT Pericellular fibrosis (% area)	3.27 ± 2.07	3.78 ± 2.55	0.536
VAT Pericellular fibrosis (% area)	2.17 ± 1.52	2.67 ± 1.67	0.418
SAT:VAT fibrosis ratio	1.15 (0.78–2.04)	1.09 (0.74–1.76)	0.808

SAT, subcutaneous adipose tissue; VAT, visceral adipose tissue. *p*-values were calculated using Welch’s *t*-test or Mann-Whitney test.

**Table 3:** Significant results of differential gene expression analysis in SAT and VAT from Ob-NAFLD vs. Ob groups.

	Ob (*n* = 24)			Ob-NAFLD (*n* = 24)				
	Median	Percentile		Median	Percentile			
Tissue Depot		25%	75%		25%	75%	FC	*p*-Value
**SAT**								
CD68	0.834	0.546	1.158	1.224	0.831	1.707	1.42	0.0162
MRC1	0.0940	0.0615	0.147	0.158	0.0939	0.223	1.61	0.0117
MSR1	0.154	0.0742	0.218	0.211	0.162	0.367	1.71	0.0171
VEGFB	0.237	0.177	0.260	0.265	0.216	0.355	1.38	0.0321
ANGPT1	0.0256	0.0165	0.0331	0.0340	0.0254	0.0477	1.41	0.0269
P16	0.0307	0.0020	0.0496	0.0060	0.0013	0.0211	0.30	0.0264
LEP	0.707	0.570	0.915	1.240	0.624	1.764	1.75	0.0353
LEPR	0.382	0.215	1.090	0.220	0.188	0.280	0.36	0.0105
PGC1A	0.0067	0.0048	0.0074	0.0048	0.0026	0.0053	0.73	0.0167
TGFB1	0.135	0.103	0.160	0.184	0.137	0.265	1.70	0.0021
UCP2	0.283	0.194	0.443	0.460	0.344	0.570	1.44	0.0024
COL1A1	0.304	0.208	0.571	0.707	0.492	0.963	1.35	0.0036
COL4A1	1.592	1.232	1.876	2.205	1.726	3.081	1.55	0.0070
COL6A1	0.0117	0.0093	0.0166	0.0392	0.0134	0.0847	9.44	0.0023
TIMP3	1.320	0.757	1.956	0.836	0.623	0.987	0.48	0.0074
**VAT**								
HIF1A	0.348	0.259	0.434	0.244	0.178	0.374	0.81	0.0373
ANGPT2	0.0273	0.0227	0.0388	0.0209	0.0130	0.0293	0.65	0.0246
P53	0.0762	0.0561	0.155	0.0507	0.0432	0.0613	0.49	0.0019
P16	0.0098	0.0026	0.0170	0.0052	0.0008	0.0096	0.35	0.0348
LEP	0.290	0.157	0.675	0.422	0.335	1.308	1.87	0.0182
MGLL	0.389	0.242	0.467	0.592	0.391	0.688	1.67	0.0038
ATG12	0.0558	0.0387	0.0707	0.0646	0.0513	0.0953	1.32	0.0309
CIDEA	0.0343	0.0262	0.0405	0.0191	0.0098	0.0348	0.63	0.0039

*p*-values were calculated using Welch’s *t*-test or Mann-Whitney test. SAT, subcutaneous adipose tissue; VAT, visceral adipose tissue; FC, fold change.

**Table 4:** Multinomial logistic regression of VAT and SAT gene expression associated with NAFLD.

Variable	B	S.E. (B)	Exp B (OR) (95% C.I)	Sig.	R2 (%)	Correct Prediction (%)	M. Sig.
MODEL 1							
SAT-TGFB1	1.397	0.642	4.04 (1.15–14.23)	0.030	45.2	80	0.000
VAT-P53	−1.400	0.634	0.25 (0.07–0.85)	0.027			
Age	−0.016	0.040	0.98 (0.91–1.06)	0.683			
MODEL 2							
SAT-TGFB1	110.07	8455.23	6.33 (0.00–>50.00)	0.790	100	100	0.000
VAT-P53	−56.76	5653.40	<0.001 (–)	0.792			
VAT-CIDEA	−390.28	13264.12	<0.001 (–)	0.777			
SAT-UCP2	264.87	9103.50	10.73 (0.00–>50.00)	0.777			
SAT-ATG5	−54.44	2596.91	<0.001 (–)	0.783			
SAT-MRC1	−97.42	7094.69	<0.001 (–)	0.789			
VAT-ANGPT2	−149.39	5819.60	<0.001 (–)	0.780			
Age	8.92	312.56	74.90 (0.00–>100.00)	0.777			

B, Beta coefficient; S.E. (B), Standard error of Beta; Exp B (OR) (95% C.I), Exponent beta with 95% confidence intervals; Sig, *p* value tested for each independent variable enter in the model; R2, Nagelkerke R Square of the model; Correct prediction (%), Percentage of patients correctly classified for NAFLD according to model; M. Sig, Model significance based on Omnibus test of the model coefficients.

## Data Availability

All data presented in this study are reported in this manuscript or available in Supplementary Material.

## Supplementary Materials

The following are available online at https://www.mdpi.com/article/10.3390/ijms221910541/s1.
