# Supplementary material for: A Distinctive NAFLD Signature in Adipose Tissue from Women with Severe Obesity
Source: Int J Mol Sci. 2021 Sep 29;22(19):10541. doi: 10.3390/ijms221910541 (PMC8509058; doi:10.3390/ijms221910541)
Supplement: Supplementary file 1 [file ijms-22-10541-s001.zip › ijms-1383221-supplementary.pdf]

Supplementary Figure 1

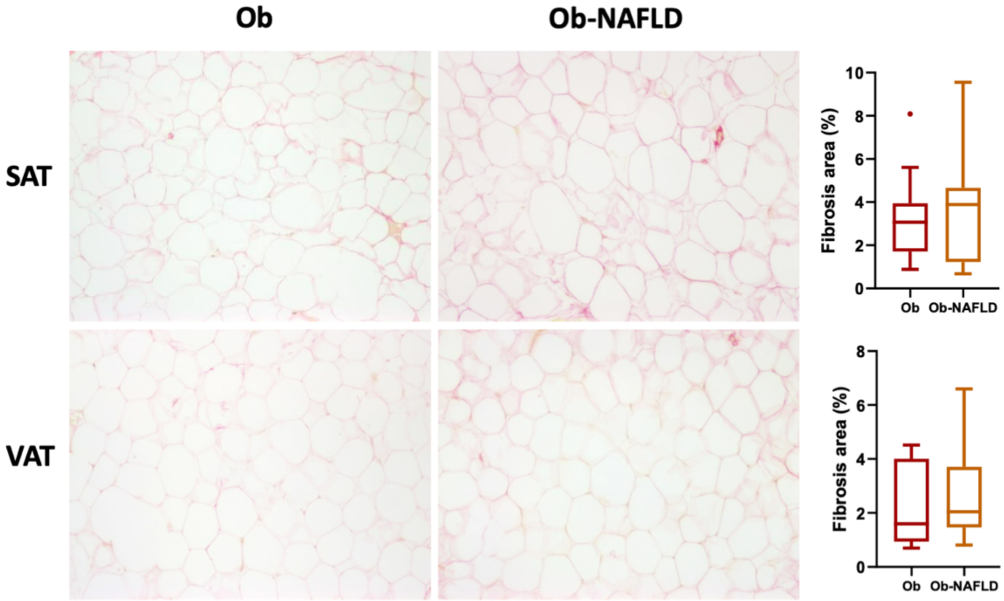

Supplementary Figure 1. Adipose tissue fibrosis. Representative images and Tukey's box plot showing histological pericelullar fibrosis in SAT and VAT. Data are presented as the ratio of fibrous tissue area stained with picrosirius red/total tissue surface. SAT, subcutaneous adipose tissue; VAT, visceral adipose tissue.

Supplementary Figure 2

SAT

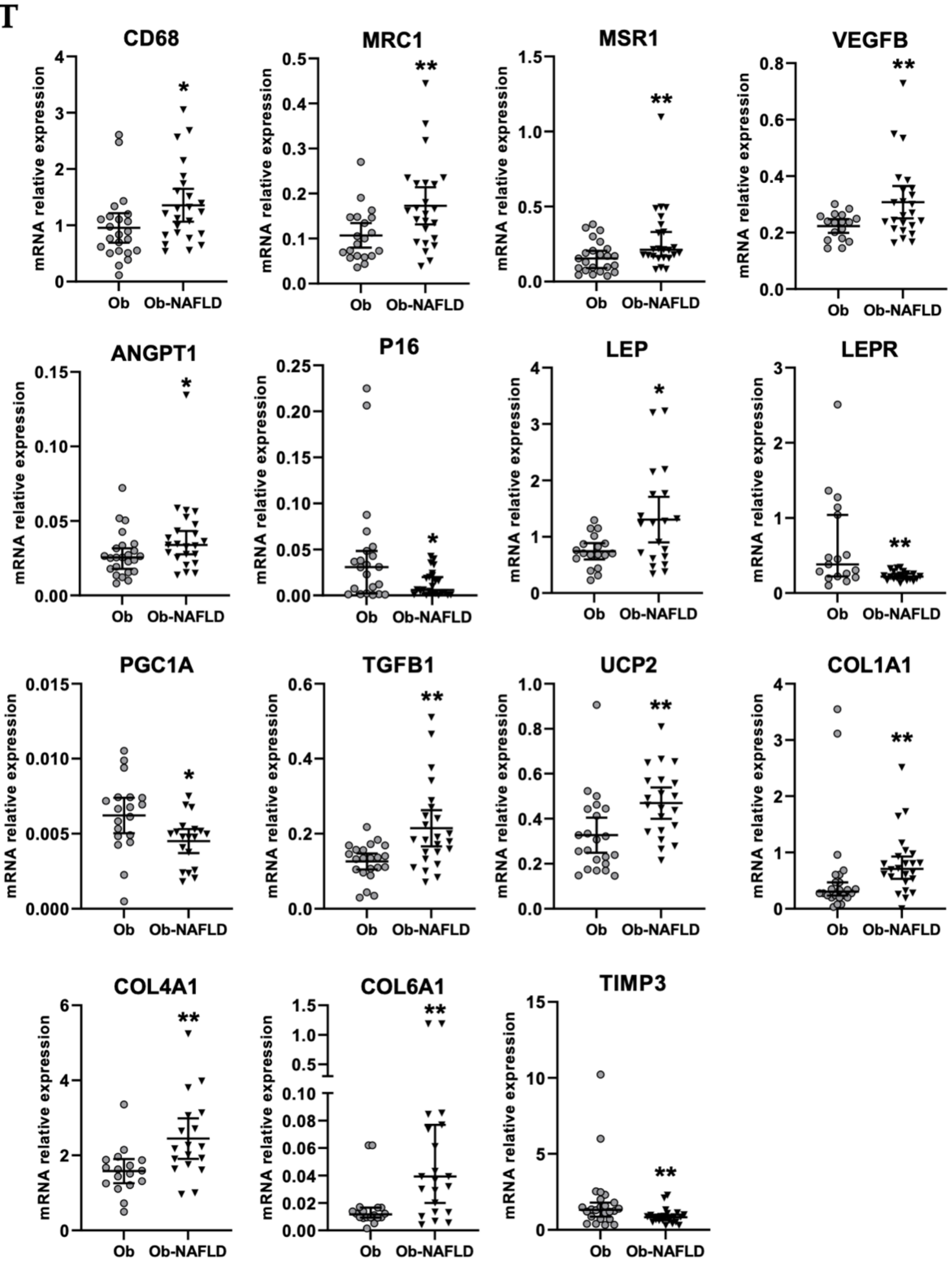

Supplementary Figure 2. Expression analysis of genes involved in WAT dysfunction in SAT. Data are expressed as mean with 95% CI or median with 95% CI and compared by Welch's t-test or Mann-Whitney test, respectively. SAT, subcutaneous adipose tissue. *= p<0.05, **= p<0.01.

Supplementary Figure 3

VAT

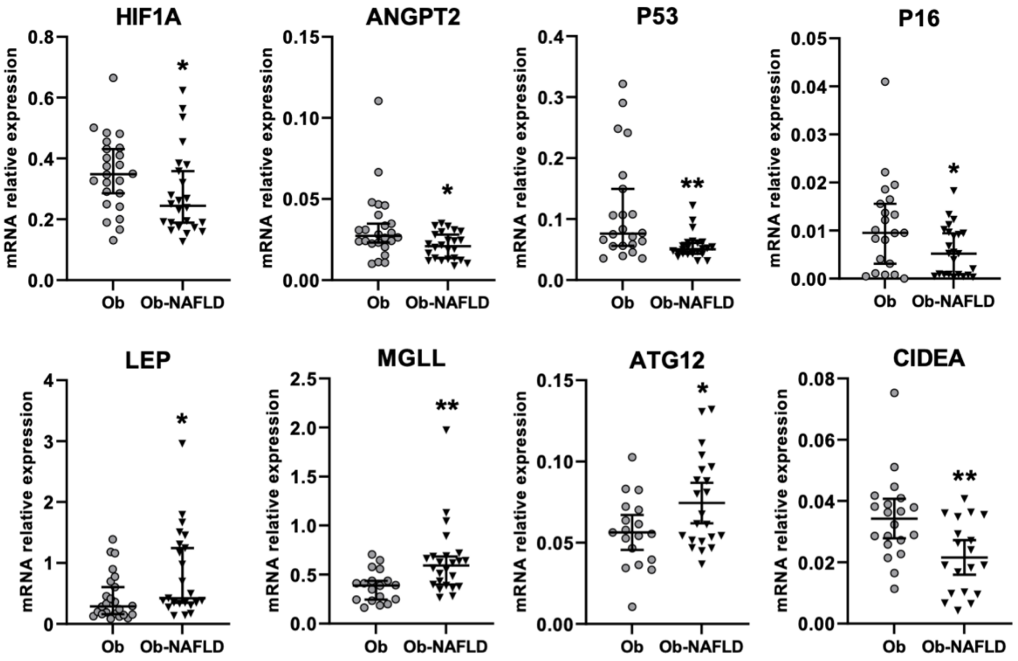

Supplementary Figure 3. Expression analysis of genes involved in WAT dysfunction in VAT. Data are expressed as mean with 95% CI or median with 95% CI and compared by Welch's t-test or Mann-Whitney test, respectively. VAT, visceral adipose tissue. *= p<0.05, **= p<0.01.

Supplementary Figure 4

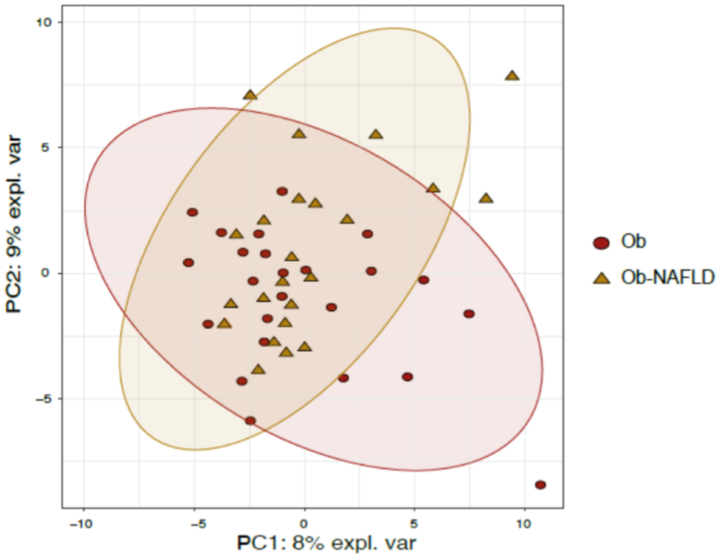

Supplementary Figure 4. **Principal component analysis comparing SAT and VAT gene expression levels between obese female patients with or without NAFLD.** Individual plot from principal component analysis based on 5 component decompositions of the gene expression matrix. Component's orthogonality was checked. Centering of data followed by scalation to equal means and standard deviation were employed. Ellipses correspond to 95% confidence intervals of groups.

Supplementary Table 1.
Descriptive statistics of the gene expression analysis performed in SAT and VAT.

	Ob (n=24)						Ob-NAFLD (n=24)						FC	p-value
	Mean	SEM	SD	Percentiles			Mean	SEM	SD	Percentiles				
				25%	50%	75%				25%	50%	75%		
SAT-CD68	0.95	0.13	0.61	0.55	0.83	1.16	1.36	0.14	0.69	0.83	1.22	1.71	1.42	0.016
SAT-CD80	0.01	0.00	0.01	0.00	0.00	0.01	0.00	0.00	0.00	0.00	0.00	0.00	0.66	0.421
SAT-CD14	0.83	0.12	0.57	0.31	0.75	0.99	0.76	0.07	0.34	0.56	0.73	0.93	0.92	0.918
SAT-MRC1	0.11	0.01	0.06	0.06	0.09	0.15	0.17	0.02	0.10	0.09	0.16	0.22	1.61	0.012
SAT-P53	0.12	0.02	0.09	0.05	0.07	0.18	0.11	0.01	0.05	0.08	0.11	0.15	0.92	0.983
SAT-MSR1	0.16	0.02	0.10	0.07	0.15	0.22	0.28	0.04	0.22	0.16	0.21	0.37	1.71	0.017
SAT-MCP1	0.70	0.29	1.21	0.09	0.26	0.64	1.12	0.42	2.04	0.20	0.29	0.82	1.61	0.204
SAT-HIF1A	0.46	0.08	0.40	0.20	0.26	0.75	0.46	0.06	0.32	0.25	0.36	0.58	0.99	0.297
SAT-IL1B	0.02	0.01	0.04	0.00	0.01	0.02	0.05	0.03	0.13	0.01	0.02	0.03	2.24	0.064
SAT-IL6	0.51	0.22	0.97	0.03	0.19	0.42	0.53	0.20	0.97	0.04	0.17	0.47	1.05	0.981
SAT-TNFA	0.00	0.00	0.00	0.00	0.00	0.00	0.00	0.00	0.00	0.00	0.00	0.00	0.54	0.237
SAT-PAI-1	0.21	0.05	0.25	0.05	0.13	0.25	0.23	0.06	0.28	0.06	0.18	0.29	1.10	0.665
SAT-ADIPOQ	4.19	0.44	2.12	2.56	3.92	5.03	6.20	0.92	4.49	3.19	5.42	7.75	1.48	0.097
SAT-VEGFA	0.07	0.01	0.07	0.03	0.04	0.13	0.04	0.00	0.01	0.03	0.03	0.04	0.53	0.328
SAT-VEGFB	0.22	0.01	0.05	0.18	0.24	0.26	0.31	0.03	0.14	0.22	0.26	0.35	1.38	0.032
SAT-VEGFR1	0.26	0.06	0.28	0.15	0.17	0.27	0.17	0.01	0.06	0.12	0.16	0.21	0.63	0.383
SAT-VEGFR2	0.08	0.01	0.04	0.06	0.10	0.11	0.11	0.01	0.04	0.08	0.10	0.13	1.33	0.153
SAT-ANGPT1	0.03	0.00	0.01	0.02	0.03	0.03	0.04	0.01	0.02	0.03	0.03	0.05	1.41	0.027
SAT-ANGPT2	0.07	0.02	0.09	0.02	0.03	0.06	0.04	0.01	0.03	0.02	0.04	0.05	0.60	0.885
SAT-PDGFRB	0.61	0.11	0.49	0.25	0.45	0.77	0.38	0.04	0.21	0.23	0.31	0.51	0.63	0.187
SAT-ADIPOR1	0.18	0.01	0.06	0.14	0.18	0.22	0.20	0.01	0.06	0.14	0.20	0.24	1.07	0.443
SAT-ADIPOR2	0.17	0.02	0.07	0.12	0.17	0.20	0.20	0.02	0.09	0.12	0.19	0.27	1.21	0.175
SAT-p16	0.04	0.01	0.06	0.00	0.03	0.05	0.01	0.00	0.01	0.00	0.01	0.02	0.30	0.026
SAT-p21	0.21	0.04	0.18	0.06	0.19	0.30	0.16	0.04	0.19	0.02	0.11	0.25	0.78	0.272
SAT-PDGFRA	0.09	0.01	0.04	0.06	0.08	0.14	0.12	0.01	0.04	0.08	0.11	0.14	1.25	0.078
SAT-ABCA1	0.52	0.15	0.68	0.17	0.27	0.64	0.44	0.06	0.30	0.24	0.30	0.55	0.83	0.588
SAT-ATG12	0.16	0.03	0.14	0.07	0.09	0.23	0.09	0.01	0.03	0.07	0.08	0.11	0.54	0.329
SAT-ATG5	0.15	0.03	0.13	0.06	0.09	0.27	0.07	0.00	0.02	0.06	0.07	0.08	0.47	0.192
SAT-ATG7	0.09	0.02	0.08	0.04	0.05	0.15	0.05	0.00	0.01	0.04	0.05	0.06	0.57	0.521
SAT-ATGL	7.00	1.54	7.05	2.17	3.23	11.71	3.75	0.51	2.28	2.42	3.31	3.84	0.54	0.639
SAT-BMP2	0.03	0.01	0.02	0.02	0.03	0.04	0.03	0.00	0.02	0.02	0.03	0.04	1.01	0.604
SAT-BMP4	0.03	0.00	0.01	0.02	0.03	0.03	0.03	0.00	0.02	0.02	0.02	0.05	1.01	0.375
SAT-DGAT2	0.41	0.06	0.22	0.22	0.35	0.63	0.55	0.10	0.43	0.22	0.37	0.82	1.35	0.691
SAT-FABP4	22.91	9.83	48.15	5.48	7.29	21.58	6.56	0.66	3.02	4.72	6.24	7.69	0.29	0.072
SAT-FASN	0.40	0.09	0.38	0.18	0.26	0.49	0.36	0.04	0.20	0.16	0.34	0.45	0.89	0.715
SAT-GLUT1	0.00	0.00	0.00	0.00	0.00	0.00	0.00	0.00	0.00	0.00	0.00	0.00	0.95	0.266
SAT-GLUT4	0.32	0.16	0.79	0.03	0.07	0.33	0.14	0.05	0.23	0.05	0.07	0.09	0.43	0.865
SAT-HSL	0.06	0.03	0.11	0.01	0.03	0.06	0.19	0.09	0.42	0.01	0.03	0.15	3.06	0.443
SAT-IRS1	0.12	0.04	0.17	0.02	0.04	0.17	0.04	0.01	0.03	0.02	0.03	0.06	0.37	0.375
SAT-KLB	0.49	0.11	0.53	0.11	0.28	0.80	0.21	0.03	0.15	0.08	0.16	0.32	0.43	0.174
SAT-LEP	0.74	0.07	0.29	0.57	0.71	0.92	1.31	0.19	0.86	0.62	1.24	1.76	1.75	0.035
SAT-LEPR	0.64	0.15	0.63	0.21	0.38	1.09	0.23	0.01	0.06	0.19	0.22	0.28	0.36	0.010

SAT-LPL	2.54	0.27	1.16	1.93	2.45	3.06	3.11	0.33	1.49	2.14	3.24	4.26	1.23	0.197
SAT-MGLL	1.64	0.21	0.83	1.13	1.58	1.96	1.29	0.17	0.72	0.74	1.12	1.68	0.79	0.104
SAT-MOGAT1	0.00	0.00	0.00	0.00	0.00	0.00	0.00	0.00	0.00	0.00	0.00	0.00	0.94	0.865
SAT-NPY1R	0.38	0.09	0.43	0.10	0.18	0.49	0.17	0.02	0.11	0.08	0.12	0.31	0.46	0.235
SAT-PGC1A	0.01	0.00	0.00	0.00	0.01	0.01	0.00	0.00	0.00	0.00	0.00	0.01	0.73	0.017
SAT-PLIN1	2.79	0.38	1.85	1.42	2.40	3.72	2.35	0.22	1.07	1.66	2.32	3.12	0.84	0.869
SAT-PLIN2	0.35	0.04	0.19	0.25	0.35	0.39	0.38	0.03	0.17	0.25	0.38	0.48	1.09	0.343
SAT-PPARG	0.61	0.07	0.35	0.42	0.50	0.71	0.52	0.03	0.15	0.44	0.52	0.62	0.84	0.821
SAT-SREBF1	0.10	0.01	0.06	0.05	0.10	0.13	0.12	0.02	0.08	0.05	0.09	0.16	1.16	0.773
SAT-TGFB1	0.13	0.01	0.05	0.10	0.13	0.16	0.21	0.02	0.11	0.14	0.18	0.26	1.70	0.002
SAT-UCP2	0.33	0.04	0.18	0.19	0.28	0.44	0.47	0.03	0.15	0.34	0.46	0.57	1.44	0.002
SAT-UCP3	0.00	0.00	0.00	0.00	0.00	0.01	0.00	0.00	0.00	0.00	0.00	0.00	0.44	0.489
SAT-LEP/ADIPOQ	0.23	0.02	0.10	0.14	0.25	0.33	0.24	0.03	0.12	0.13	0.28	0.36	1.04	0.682
SAT-COL1A1	0.59	0.18	0.87	0.21	0.30	0.57	0.80	0.11	0.55	0.49	0.71	0.96	1.35	0.004
SAT-COL3A1	1.82	0.60	2.83	0.12	0.86	1.94	0.94	0.21	0.93	0.22	0.41	1.36	0.52	0.676
SAT-COL4A1	1.58	0.15	0.63	1.23	1.59	1.88	2.45	0.26	1.08	1.73	2.21	3.08	1.55	0.007
SAT-COL5A1	0.32	0.08	0.36	0.12	0.20	0.32	0.24	0.03	0.12	0.14	0.21	0.34	0.75	0.916
SAT-COL6A1	0.02	0.00	0.02	0.01	0.01	0.02	0.16	0.07	0.33	0.01	0.04	0.08	9.44	0.002
SAT-COL6A3	0.43	0.09	0.42	0.24	0.33	0.41	0.48	0.05	0.24	0.29	0.40	0.64	1.10	0.061
SAT-MMP2	1.15	0.24	1.13	0.57	0.79	1.11	0.95	0.10	0.41	0.55	0.93	1.18	0.83	0.401
SAT-MMP14	0.30	0.07	0.32	0.14	0.21	0.29	0.23	0.02	0.08	0.19	0.24	0.27	0.78	0.430
SAT-MMP15	0.03	0.01	0.03	0.02	0.03	0.04	0.04	0.00	0.02	0.02	0.04	0.04	1.10	0.282
SAT-TIMP1	0.26	0.06	0.26	0.08	0.19	0.30	0.18	0.03	0.13	0.12	0.14	0.22	0.69	0.367
SAT-TIMP2	1.11	0.30	1.33	0.30	0.40	2.06	1.12	0.19	0.90	0.36	0.78	1.96	1.01	0.263
SAT-TIMP3	1.85	0.43	2.12	0.76	1.32	1.96	0.89	0.10	0.47	0.62	0.84	0.99	0.48	0.007
SAT-HYAL1	0.03	0.01	0.03	0.02	0.02	0.03	0.02	0.00	0.01	0.01	0.02	0.03	0.75	0.828
SAT-LOX	0.27	0.07	0.33	0.13	0.18	0.33	0.25	0.03	0.13	0.15	0.22	0.33	0.92	0.490
SAT-BGN	0.32	0.05	0.21	0.16	0.26	0.51	0.44	0.08	0.39	0.28	0.38	0.48	1.38	0.122
SAT-LOXL4	0.01	0.00	0.01	0.01	0.01	0.01	0.02	0.00	0.01	0.01	0.01	0.02	1.40	0.089
SAT-HAS1	0.01	0.00	0.01	0.00	0.00	0.01	0.02	0.01	0.06	0.00	0.00	0.01	3.40	0.856
SAT-HAS2	0.06	0.02	0.11	0.01	0.02	0.04	0.03	0.01	0.03	0.01	0.01	0.03	0.43	0.086
SAT-ELN	0.44	0.09	0.46	0.15	0.26	0.55	0.40	0.05	0.24	0.25	0.31	0.48	0.92	0.375
SAT-FN1	0.85	0.24	1.10	0.33	0.53	0.66	0.88	0.19	0.80	0.39	0.61	1.25	1.03	0.403
SAT-CTGF	0.96	0.20	0.99	0.38	0.65	1.16	1.00	0.16	0.78	0.42	0.73	1.69	1.04	0.621
SAT-F13A1	0.33	0.04	0.16	0.17	0.32	0.47	0.57	0.10	0.41	0.21	0.46	0.89	1.72	0.092
SAT-ITGB2	0.15	0.03	0.13	0.07	0.11	0.22	0.28	0.07	0.32	0.13	0.18	0.33	1.84	0.074
SAT-SPP1	0.45	0.07	0.36	0.13	0.34	0.74	0.66	0.12	0.59	0.21	0.46	1.24	1.46	0.194

	Ob (n=24)						Ob-NAFLD (n=24)						FC	p-value
	Mean	SEM	SD	Percentiles			Mean	SEM	SD	Percentiles				
				25%	50%	75%				25%	50%	75%		
VAT-CD68	0.78	0.12	0.55	0.46	0.64	0.86	0.86	0.08	0.39	0.55	0.75	1.04	1.10	0.109
VAT-CD14	0.53	0.06	0.27	0.35	0.49	0.66	0.50	0.04	0.20	0.31	0.44	0.66	0.94	0.826
VAT-MRC1	0.14	0.01	0.06	0.09	0.13	0.17	0.15	0.01	0.06	0.10	0.14	0.19	1.13	0.305
VAT-P53	0.11	0.02	0.09	0.06	0.08	0.16	0.06	0.00	0.02	0.04	0.05	0.06	0.49	0.002
VAT-MSR1	0.10	0.02	0.06	0.07	0.10	0.11	0.11	0.01	0.05	0.08	0.10	0.15	1.11	0.328
VAT-MCP1	0.98	0.24	1.00	0.14	0.63	1.39	0.78	0.17	0.85	0.19	0.52	0.94	0.79	0.491
VAT-HIF1A	0.35	0.03	0.12	0.26	0.35	0.43	0.29	0.03	0.14	0.18	0.24	0.37	0.81	0.037

VAT-IL1B	0.27	0.09	0.39	0.01	0.08	0.28	0.19	0.07	0.36	0.01	0.04	0.23	0.72	0.494
VAT-IL6	0.43	0.14	0.62	0.05	0.21	0.58	0.27	0.07	0.34	0.04	0.12	0.41	0.62	0.423
VAT-TNFA	0.00	0.00	0.00	0.00	0.00	0.00	0.00	0.00	0.01	0.00	0.00	0.00	1.34	0.054
VAT-PAI-1	0.23	0.06	0.27	0.08	0.14	0.30	0.13	0.03	0.14	0.04	0.09	0.16	0.56	0.059
VAT-ADIPOQ	3.36	0.23	1.03	2.71	3.28	4.24	3.77	0.40	1.94	2.38	3.47	4.82	1.12	0.399
VAT-VEGFA	0.05	0.01	0.03	0.04	0.05	0.07	0.04	0.00	0.02	0.03	0.04	0.06	0.80	0.112
VAT-VEGFB	0.22	0.02	0.08	0.17	0.19	0.25	0.24	0.02	0.08	0.19	0.23	0.31	1.10	0.362
VAT-VEGFR1	0.19	0.02	0.09	0.12	0.19	0.24	0.15	0.01	0.07	0.12	0.13	0.17	0.82	0.186
VAT-VEGFR2	0.12	0.01	0.05	0.09	0.10	0.15	0.12	0.01	0.04	0.09	0.12	0.14	1.00	0.582
VAT-ANGPT1	0.03	0.00	0.02	0.02	0.03	0.04	0.02	0.00	0.01	0.02	0.02	0.03	0.73	0.058
VAT-ANGPT2	0.03	0.00	0.02	0.02	0.03	0.04	0.02	0.00	0.01	0.01	0.02	0.03	0.65	0.025
VAT-PDGFRB	0.18	0.02	0.09	0.12	0.15	0.22	0.14	0.01	0.06	0.09	0.13	0.19	0.78	0.141
VAT-ADIPOR1	0.17	0.01	0.05	0.12	0.17	0.20	0.19	0.02	0.08	0.14	0.17	0.20	1.09	0.674
VAT-ADIPOR2	0.16	0.01	0.06	0.11	0.17	0.19	0.18	0.02	0.08	0.13	0.15	0.25	1.16	0.270
VAT-p16	0.02	0.01	0.03	0.00	0.01	0.02	0.01	0.00	0.01	0.00	0.01	0.01	0.35	0.035
VAT-p21	0.13	0.04	0.17	0.03	0.08	0.17	0.07	0.01	0.07	0.01	0.04	0.11	0.50	0.099
VAT-PDGFRA	0.13	0.01	0.04	0.10	0.12	0.15	0.13	0.01	0.06	0.10	0.12	0.15	1.06	0.979
VAT-FASN	0.36	0.06	0.23	0.19	0.24	0.54	0.32	0.04	0.17	0.21	0.32	0.37	0.89	0.959
VAT-LPL	2.44	0.28	1.40	1.68	2.22	2.98	2.30	0.26	1.24	1.54	2.07	3.09	0.94	0.848
VAT-MOGAT1	0.00	0.00	0.01	0.00	0.00	0.00	0.00	0.00	0.00	0.00	0.00	0.00	0.82	0.640
VAT-DGAT2	0.34	0.05	0.22	0.21	0.33	0.38	0.42	0.06	0.30	0.22	0.40	0.60	1.22	0.278
VAT-PLIN1	1.85	0.27	1.34	0.84	1.53	2.26	1.83	0.13	0.63	1.31	1.93	2.36	0.99	0.398
VAT-MGLL	0.38	0.04	0.16	0.24	0.39	0.47	0.64	0.07	0.36	0.39	0.59	0.69	1.67	0.004
VAT-ATGL	2.97	0.37	1.70	1.60	2.47	4.07	3.25	0.31	1.47	1.97	3.22	4.12	1.10	0.318
VAT-GLUT1	0.01	0.00	0.01	0.00	0.00	0.01	0.01	0.00	0.01	0.00	0.01	0.01	0.93	0.590
VAT-GLUT4	0.08	0.03	0.15	0.03	0.04	0.06	0.05	0.01	0.04	0.02	0.04	0.07	0.71	0.787
VAT-IRS1	0.02	0.00	0.01	0.01	0.02	0.03	0.03	0.00	0.02	0.01	0.03	0.03	1.54	0.093
VAT-PPARG	0.34	0.04	0.20	0.21	0.29	0.46	0.44	0.05	0.24	0.29	0.39	0.54	1.28	0.152
VAT-LEP	0.44	0.08	0.38	0.16	0.29	0.68	0.82	0.15	0.71	0.34	0.42	1.31	1.87	0.018
VAT-LEPR	0.27	0.02	0.08	0.22	0.25	0.27	0.28	0.02	0.11	0.20	0.28	0.35	1.05	0.565
VAT-PGC1A	0.01	0.00	0.00	0.01	0.01	0.01	0.01	0.00	0.01	0.01	0.01	0.01	1.34	0.235
VAT-UCP2	0.36	0.05	0.22	0.21	0.29	0.42	0.44	0.04	0.19	0.28	0.40	0.51	1.22	0.059
VAT-UCP3	0.00	0.00	0.00	0.00	0.00	0.00	0.00	0.00	0.00	0.00	0.00	0.00	1.66	0.072
VAT-ATG5	0.05	0.00	0.02	0.03	0.06	0.06	0.06	0.01	0.03	0.05	0.06	0.07	1.31	0.056
VAT-ATG7	0.04	0.00	0.01	0.03	0.04	0.05	0.04	0.00	0.01	0.03	0.04	0.05	0.94	0.512
VAT-ATG12	0.06	0.01	0.02	0.04	0.06	0.07	0.07	0.01	0.03	0.05	0.06	0.10	1.32	0.031
VAT-KLB	0.09	0.01	0.05	0.05	0.08	0.12	0.11	0.01	0.06	0.05	0.11	0.14	1.19	0.358
VAT-BMP2	0.03	0.00	0.01	0.02	0.03	0.04	0.04	0.00	0.02	0.02	0.03	0.05	1.27	0.138
VAT-BMP4	0.04	0.00	0.02	0.03	0.03	0.05	0.05	0.01	0.02	0.03	0.04	0.06	1.26	0.182
VAT-ABCA1	0.16	0.01	0.06	0.12	0.16	0.21	0.18	0.02	0.09	0.13	0.16	0.24	1.15	0.579
VAT-UCP1	0.00	0.00	0.00	0.00	0.00	0.00	0.00	0.00	0.01	0.00	0.00	0.00	0.83	0.419
VAT-HSL	0.18	0.04	0.19	0.03	0.09	0.31	0.31	0.06	0.28	0.04	0.26	0.51	1.73	0.181
VAT-SREBF1	0.05	0.00	0.02	0.03	0.05	0.06	0.07	0.01	0.05	0.03	0.07	0.10	1.46	0.096
VAT-FGFR1	0.19	0.02	0.09	0.13	0.19	0.22	0.17	0.02	0.08	0.10	0.18	0.22	0.86	0.483
VAT-FABP4	4.95	0.92	4.13	2.20	4.03	6.49	3.51	0.47	2.21	1.57	3.75	5.35	0.71	0.199
VAT-PRDM16	0.00	0.00	0.00	0.00	0.00	0.00	0.00	0.00	0.00	0.00	0.00	0.00	0.80	0.172
VAT-CIDEA	0.03	0.00	0.01	0.03	0.03	0.04	0.02	0.00	0.01	0.01	0.02	0.03	0.63	0.004
VAT-PGC1B	0.01	0.00	0.00	0.01	0.01	0.01	0.01	0.00	0.00	0.01	0.01	0.01	1.11	0.522
VAT-ADRB3	0.00	0.00	0.00	0.00	0.00	0.00	0.00	0.00	0.01	0.00	0.00	0.01	1.49	0.857

VAT-CD80	0.00	0.00	0.00	0.00	0.00	0.00	0.00	0.00	0.00	0.00	0.00	0.00	0.92	0.692
VAT-ADRB1	0.03	0.00	0.01	0.02	0.02	0.04	0.03	0.00	0.02	0.02	0.03	0.04	1.13	0.719
VAT-SOD2	1.69	0.33	1.32	0.71	1.14	2.74	2.00	0.33	1.33	0.93	1.74	2.48	1.18	0.386
VAT-LEP/ADIPOQ	0.13	0.02	0.10	0.06	0.10	0.17	0.26	0.04	0.21	0.10	0.20	0.41	1.93	0.046
VAT-COL1A1	0.46	0.05	0.16	0.28	0.48	0.60	0.35	0.04	0.17	0.20	0.36	0.42	0.75	0.071
VAT-COL4A1	0.93	0.12	0.51	0.53	0.80	1.24	1.02	0.13	0.49	0.64	0.81	1.22	1.10	0.509
VAT-COL6A1	0.23	0.11	0.44	0.00	0.09	0.30	0.19	0.05	0.19	0.01	0.15	0.33	0.82	0.558
VAT-COL6A3	0.15	0.02	0.09	0.10	0.15	0.19	0.17	0.02	0.10	0.10	0.17	0.21	1.10	0.339
VAT-MMP15	0.02	0.00	0.01	0.01	0.01	0.02	0.02	0.00	0.01	0.01	0.02	0.03	1.17	0.293
VAT-BGN	0.20	0.01	0.06	0.15	0.19	0.23	0.20	0.01	0.06	0.16	0.20	0.25	1.01	0.917
VAT-FN1	0.34	0.06	0.23	0.16	0.29	0.50	0.41	0.12	0.49	0.13	0.27	0.39	1.20	0.719
VAT-TGFB1	0.13	0.01	0.06	0.10	0.12	0.15	0.12	0.01	0.04	0.09	0.11	0.13	0.92	0.453
VAT-TIMP2	0.65	0.16	0.65	0.34	0.43	0.66	0.80	0.10	0.46	0.42	0.70	0.98	1.23	0.070
VAT-LOXL4	0.00	0.00	0.00	0.00	0.00	0.01	0.01	0.00	0.00	0.00	0.01	0.01	1.18	0.265
VAT-HAS1	0.19	0.04	0.18	0.06	0.11	0.26	0.16	0.04	0.19	0.03	0.10	0.20	0.82	0.361
VAT-F13A1	0.35	0.05	0.23	0.19	0.33	0.43	0.43	0.04	0.15	0.31	0.39	0.50	1.24	0.054
VAT-NPY1R	0.09	0.03	0.13	0.04	0.06	0.08	0.07	0.02	0.07	0.03	0.06	0.10	0.83	0.887

P-values were calculated using Welch's t-test or Mann-Whitney test. SAT, subcutaneous adipose tissue; VAT, visceral adipose tissue; SEM, standard error of the mean; SD, standard deviation; FC, fold change.

Supplementary Table 2. Gene weight in the component employed for the signature model.

Component 1		Component 2	
**Gene**	**value.var**	**Gene**	**value.var**
SAT-TGFB1	-0.506	VAT-COL6A3	0.473
VAT-P53	0.458	VAT-HIF1A	-0.331
VAT-CIDEA	0.278	VAT-IL1B	-0.305
SAT-UCP2	-0.261	VAT-p21	-0.299
SAT-ATG5	0.235	SAT-ANGPT1	0.297
VAT-MGLL	-0.225	VAT-IL6	-0.277
SAT-MRC1	-0.203	VAT-PAI-1	-0.265
VAT-ANGPT2	0.201	VAT-BMP4	0.235
SAT-LEPR	0.175	SAT-CD80	-0.183
SAT-p16	0.150	VAT-DGAT2	0.182
SAT-ATG12	0.147	SAT-HIF1A	-0.174
SAT-COL4A1	-0.138	VAT-ATG7	-0.174
SAT-MSR1	-0.127	SAT-PLIN1	0.143
SAT-VEGFA	0.118	SAT-KLB	-0.134
VAT-LEP	-0.117	VAT-MCP1	-0.111
VAT-ANGPT1	0.117	SAT-IRS1	-0.082
SAT-LEP	-0.111	VAT-MMP15	0.050
SAT-KLB	0.088	SAT-COL5A1	-0.039
SAT-VEGFB	-0.081	SAT-TNFA	-0.039
SAT-UCP3	0.075	SAT-ABCA1	-0.037
SAT-TIMP3	0.074	VAT-UCP2	0.032
SAT-PGC1A	0.071	SAT-LEP	0.028
VAT-IRS1	-0.071	SAT-COL6A3	-0.024
SAT-CD68	-0.039	VAT-FN1	0.017
SAT-VEGFR2	-0.015	VAT-F13A1	0.013

SAT, subcutaneous adipose tissue; VAT, visceral adipose tissue; Value.var, value variance.

Supplementary Table 3. Spearman correlation between gene expression levels of those genes involved in Multinomial logistic regression model

			SAT-TGFB1	VAT-P53	VAT-CIDEA	SAT-UCP2	SAT-ATG5	SAT-MRC1	VAT-ANGPT2
Spearman's rho	SAT-TGFB1	Correlation Coefficient	1.000						
		Sig. (2-tailed)							
	VAT-P53	Correlation Coefficient	-0.323	1.000					
		Sig. (2-tailed)	0.030						
	VAT-CIDEA	Correlation Coefficient	-0.188	0.464	1.000				
		Sig. (2-tailed)	0.264	0.003					
	SAT-UCP2	Correlation Coefficient	0.264	-0.436	-0.211	1.000			
		Sig. (2-tailed)	0.096	0.004	0.203				
	SAT-ATG5	Correlation Coefficient	0.191	-0.072	0.046	0.094	1.000		
		Sig. (2-tailed)	0.226	0.651	0.779	0.548			
	SAT-MRC1	Correlation Coefficient	0.809	-0.084	-0.048	0.271	0.213	1.000	
		Sig. (2-tailed)	0.000	0.589	0.779	0.095	0.187		
	VAT-ANGPT2	Correlation Coefficient	-0.291	0.345	0.192	-0.157	-0.239	-0.173	1.000
		Sig. (2-tailed)	0.050	0.019	0.241	0.314	0.118	0.262	

SAT, subcutaneous adipose tissue; VAT, visceral adipose tissue; Sig., significance.

Supplementary Table 4. List of oligonucleotides

**GENE**	**SEQUENCE**	**(5'-3')**
ABCA1	F	GGAGGCCAGAATGACATCTTAG
ABCA1	R	TTTCCAGCCCCATTAACTCC
ACOX1	F	ACCATTGCCATCCGATACAG
ACOX1	R	GGTCTCCTTCATGTATGCGC
ADFP/PLIN2	F	AGTATCCCTACCTGAAGTCTGTG
ADFP/PLIN2	R	CCCCTTACAGGCATAGGTATTG
ADIPOQ	F	ACAATGACTCCACCTTCACAG
ADIPOQ	R	TTCCTAACCGTACTGAAAGCC
ADRB1	F	CCGGGAACAGGAACACAC
ADRB1	R	GAAAGCAAAAGGAAATATGTCTTGA
ADRB3	F	TTTTCTAAACCCCAGCCTTG
ADRB3	R	CACGGCACCTGGACACTAC
ANGPT1	F	TTAAAGGACTTACAGGGACAGC
ANGPT1	R	GACCACATGCATCAAACCAC
ANGPT2	F	CCACGAGACTTGAACTTCAGC
ANGPT2	R	TGTGCTTGTCTTCCATAGCTAG
APOE	F	CAGCGACAATCACTGAACG
APOE	R	GTGAATCTTTATTAAACTAGGGTCCAC
ATG12	F	AATCAGTCCTTTGCTCCTTCC
ATG12	R	GCAAGTTGATTTTCTTTGTGGTTC
ATG5	F	AGCAACTCTGGATGGGATTG
ATG5	R	AGGTCTTTCAGTCGTTGTCTG
ATG7	F	TTTTGCTATCCTGCCCTCTG
ATG7	R	GCTGTGACTCCTTCTGTTTGAC
ATGL	F	CACTTCAACTCCAAGGACGAG
ATGL	R	CTCATAGAGTGGCAGGTTGTC
BMP2	F	CTATCAGGACATGGTTGTGGAG
BMP2	R	GGGAAATATTAAAGTGTCAACTGGG
BMP4	F	TGGCTGTCAAGAATCATGGAC
BMP4	R	CCCGTCTCAGGTATCAAACTAG
CD14	F	CAGAGGTTCGGAAGACTTATCG
CD14	R	TTCGGAGAAGTTGCAGACG
CD206/MRC1	F	GCAAAGTGGATTACGTGTCTTG
CD206/MRC1	R	CTGTTATGTCGCTGGCAAATG
CD68	F	ATGGCGGTGGAGTACAATG
CD68	R	TGGACAGCTGGTGAAAGAATG
CD80	F	CCATCCAAGTGTCCATACCTC
CD80	F	CTCACTTCTGTTCAGGTGTTATCCA

CD80	R	GCCAGCTCTTCAACAGAAAC
CD80	R	TCCTTTTGCCAGTAGATGCGA
CD86	F	ACATTCTCTTTGTGATGGCCTTC
CD86	R	TGCAGTCTCATTGAAATAAGCTTGA
CIDEA	F	GGCAGGTTCACGTGTGGATA
CIDEA	R	GAAACACAGTGTTTGGCTCAAGA
CPT1A	F	TCCAGTTGGCTTATCGTGGTG
CPT1A	R	CTAACGAGGGGTCGATCTTGG
DGAT2	F	TCCGAATGCCTGTGTTGAG
DGAT2	R	CAAATAGTCTATGGTGTCCCGG
FABP4	F	CATGTGCAGAAATGGGATGG
FABP4	R	AACTTCAGTCCAGGTCAACG
FASN	F	CAGAGTCGGAGAACTTGCAG
FASN	R	GGAGGCATCAAACCTAGACAG
HIF1A	F	AAGAACTTTTAGGCCGCTCA
HIF1A	R	CAACCCAGACATATCCACCTC
HSL/LIPE	F	TCATCTCCATCGACTACTCCC
HSL/LIPE	R	AGATTCGTTCCCCTGTTGAG
IL6	F	CAACCTGAACCTTCCAAAGATG
IL6	R	ACCTCAAACTCCAAAAGACCAG
IRS1	F	TCTGCTCAGCGTTGGTG
IRS1	R	GTGCATGCTCTTGGGTTTG
KLB	F	CATGGGTATGGGACAGGTATG
KLB	R	TCTGATGTGGGCGGAAATG
LEP	F	GCTTCAGGCTACTCCACAG
LEP	R	CCTTCCCTTAACGTAGTCCTTG
LEPR	F	TCAACCAGTACAATCCAGTCAC
LEPR	R	TTTGGGCTCAGATATGGGATG
LPL	F	GGACTGAGAGTGAAACCCATAC
LPL	R	GGAAGGAGTAGGTCTTATTTGTGG
MCP1/CCL2	F	CCTCCAGCATGAAAGTCTCTG
MCP1/CCL2	R	TCTGCACTGAGATCTTCCTATTG
GENE	SEQUENCE	(5'-3')
MGLL	F	AGCATGCCAGAGGAAAGTTC
MGLL	R	ATGGGACACAAAGATGAGGG
MOGAT1	F	GAAAGCCATCCACACTGTTG
MOGAT1	R	GCCATACTTTCCTTTGTGTTCC
MSR1	F	ATCTGTGAAATTTGATGCTCGC
MSR1	R	CCAATGAGAGGGATGAGAACTG

P16/CDKN2A	F	GATGTCGCACGGTACCTG
P16/CDKN2A	R	TCTCTGGTTCTTTCAATCGGG
P21/CDKN1A	F	GAACTTCGACTTTGTCACCGAGAC
P21/CDKN1A	R	TGGAGTGGTAGAAATCTGTCATGCT
P53/TP53	F	CAGCACATGACGGAGGTTGT
P53/TP53	R	TCATCCAAATACTCCACACGC
PAI-1/SERPINE1	F	GTGGACTTTTCAGAGGTGGAG
PAI-1/SERPINE1	R	GAAGTAGAGGGCATTCACCAG
PDGFRA	F	TTCCTCTGCCTGACATTGAC
PDGFRA	R	GTCTTCAATGGTCTCGTCCTC
PDGFRB	F	ATGTGACGGAGAGTGTGAATG
PDGFRB	R	GCAGCTCAGCAAATTGTAGTG
PGC1B	F	GTACATTCAAAATCTCTCCAGCGACAT
PGC1B	R	GAGGGCTCGTTGCGCTTCCTCAGGGC
PLIN1	F	CATTGAGAAGGTGGTGGAGTAC
PLIN1	R	GTGTATCGAGAGAGGGTGTTG
PLIN2	F	AGTATCCCTACCTGAAGTCTGTG
PLIN2	R	CCCCTTACAGGCATAGGTATTG
PPARA	F	CTATCATTTGCTGTGGAGATCG
PPARA	R	AAGATATCGTCCGGGTGGTT
PPARG	F	GTCGGTTTCAGAAATGCCTTG
PPARG	R	GCTGGTCGATATCACTGGAG
PPARGC1A/PGC1A	F	CAGGCAGTAGATCCTCTTCAAG
PPARGC1A/PGC1A	R	TCCTCGTAGCTGTCATACCTG
PRDM16	F	CACGAGTGCAAGGACTGC
PRDM16	R	TGTGGATGACCATGTGCTG
RPL6	F	CCTTAATTCTCTTTCCCATCTTGC
RPL6	R	TTCTTGGCTTCGGGTTTCTT
SDHA	F	TGGTTGTCTTTGGTCGGG
SDHA	R	GCGTTTGGTTTAATTGGAGGG
SLC2A1/GLUT1	F	TCATCGTGGCTGAACTCTTC
SLC2A1/GLUT1	R	GATGAAGACGTAGGGACCAC
SLC2A4/GLUT4	F	ACTGGACGAGCAACTTCATC
SLC2A4/GLUT4	R	GAGGACCGCAAATAGAAGGAA
SOD2	F	GACAAACCTCAGCCCTAACG
SOD2	R	GAAACCAAGCCAACCCCAAC
SREBF1	F	TTCTGACAGCCATGAAGACAG
SREBF1	R	CCGCATCTACGACCAGTG
TGFB1	F	TTGATGTCACCGGAGTTGTG

TGFB1	R	GTAGTGAACCCGTTGATGTCC
TNFA	F	AGGTCTACTTTGGGATCATTGC
TNFA	R	GAAGAGGTTGAGGGTGTCTG
UCP1	F	GGACTACTCCCAATCTGATGAG
UCP1	R	AAATCCAGCGATAAGAGCCG
UCP2	F	TCCTGAAAGCCAACCTCATG
UCP2	R	GGCAGAGTTCATGTATCTCGTC
UCP3	F	AGAAAATACAGCGGGACTATGG
UCP3	R	CTTGAGGATGTCGTAGGTCAC
VEGFA	F	AGTCCAACATCACCATGCAG
VEGFA	R	TTCCCTTTCCTCGAACTGATTT
VEGFB	F	CTTAGAGCTCAACCCAGACAC
VEGFB	R	ACCCTGCTGAGTCTGAAAAG
VEGFR1	F	TCCCTCAACCTACAATCAAGTG
VEGFR1	R	GCTCTCAATTCTGTTTCCCATG
VEGFR2	F	CATTTCAAAGGAGAAGCAGAGC
VEGFR2	R	GAGGAATGGCATAGACCGTAC

F: Forward. R: Reverse
